# Diagnostic Value of Cytology in Pancreatic Endoscopic Ultrasound-Guided Fine Needle Aspiration: Accuracy in Common Epithelial Pancreatobiliary Tumors and the Role of Cell Block Analysis 

**DOI:** 10.30699/ijp.2024.2036290.3329

**Published:** 2025-01-10

**Authors:** Naser Rakhshani, Neda Soleimani, Sanaz Aghaei, Rasha Barakat, Ali Mohammad Keshtvarz Hesam Abadi

**Affiliations:** 1 *Department of Pathology, Gastrointestinal and liver disease Research center, Firoozgar Hospital, Iran University of Medical Sciences, Tehran, Iran*; 2 *Department of Pathology, Shiraz Medical School, Shiraz University of Medical Sciences, Shiraz, Iran*; 3 *Department of Pathology, Iran Medical School, Iran University of Medical Sciences, Tehran, Iran*; 4 *Department of Biostatistics, Shiraz University of Medical Sciences, Shiraz, Iran *

**Keywords:** Accuracy, Cytology, EUS-FNA, Pancreatic adenocarcinoma, Pancreatobiliary

## Abstract

**Background & Objective:**

Limited literature describes the accuracy of endoscopic ultrasonography–fine needle aspiration (EUS-FNA) cytology in various types of pancreatic epithelial tumors, and this underscores the usefulness of cell blocks, and highlights potential diagnostic pitfalls.

**Methods:**

This study included 108 patients who underwent EUS-FNA pancreatobiliary cytology followed by surgery. Age, gender, tumor location, tumor size, presence or absence of a cell block, cytologic and pathologic diagnoses, and histologic tumor grade were recorded. Cytologic and pathologic slides were examined, and the cytologic accuracy was determined by comparing cytologic with the histopathologic results as the gold standard. Additionally, the impact of cell block on the cytologic accuracy was assessed.

**Results:**

EUS-FNA cytology showed an overall accuracy of 80%, a sensitivity of 90%, and a false-positive rate below 1%. Pancreatic ductal adenocarcinomas (PDAs) accounted for 65% of cases, followed by neuroendocrine tumors (NETs), solid pseudopapillary neoplasms (SPNs), mucinous cystic neoplasms (MCNs), and chronic pancreatitis. Diagnostic accuracy was higher for PDA and SPN than for NET and MCN and significantly improved to 100% in cases with a cell block.

**Conclusion:**

Combining pancreatobiliary cytology with a cell block significantly enhances diagnostic accuracy, reaching 100%. Moreover, poorly differentiated PDAs and well-differentiated organoid-type tumors, such as NETs and SPNs, demonstrate higher diagnostic accuracy.

## Introduction

Neoplasms of the pancreas encompass a broad spectrum and are classified according to their lines of cellular differentiation (ductal, acinar, neuroendocrine, or others) as well as their gross configuration (solid, cystic, or intraductal). Nearly 90% of adult pancreatic neoplasms are pancreatic ductal adenocarcinomas (PDAs) or closely related subtypes. Cystic and intraductal neoplasms, neuroendocrine tumors (NETs), acinar cell carcinoma, and other less common entities constitute the remainder, in that order. Mesenchymal and hematolymphoid neoplasms are extremely rare (1, 2).

Pancreatic cancer is the seventh leading cause of cancer-related deaths worldwide. From 1990 to 2019, the age-standardized incidence rate (ASIR) increased by +25.02% and +26.35% in males and females, respectively, indicating that pancreatic cancer represents a substantial public health burden (3–5). Due to the pancreas’s distinctive anatomical position, pancreatic lesions are typically identified at advanced stages when clinical symptoms become apparent. Despite major advances in medical technology and notable improvements in cancer survival overall, PDA remains highly lethal, with a 5-year survival rate below 5% and challenges in early detection. Other epithelial tumors such as NET, mucinous cystic neoplasm (MCN), and solid pseudopapillary neoplasm (SPN) have substantially better prognoses, with 5-year survival rates of 95%, 96.6%, and 97%, respectively (5–13).

Since different pancreatic masses require distinct management strategies—such as surgical resection with or without neoadjuvant therapy for PDA and surgical resection for NET and SPN—an accurate preoperative diagnosis is crucial.. Moreover, in cases like pancreatitis, lymphoma, or benign tumors, surgery can be avoided in favor of a conservative management (14–17).

Tumor markers, including CA-19-9 and carcinoembryonic antigen (CEA), have limited diagnostic utility. Abdominal ultrasound also has limited sensitivity and specificity, serving primarily as a precursor to computed tomography (CT). Triphasic contrast-enhanced abdominal CT visualizes the tumor in relation to adjacent arteries and organs, offering sensitivity, specificity, and diagnostic accuracy of 81.4%, 43%, and 83.3%, respectively. Magnetic resonance imaging (MRI) demonstrates a similar accuracy rate of 89.1% (18–20). However, endoscopic ultrasonography (EUS) has gained wider acceptance and applicability in clinical practice by allowing simultaneous fine needle aspiration (FNA) or biopsy (FNB) for subsequent pathological diagnosis (21–23).

EUS-FNA has limitations, including the inability to obtain histologic architecture, perform immunohistochemical analysis or molecular profiling, and reliance on the expertise of the endoscopist and cytopathologist (24, 25). Earlier clinical trials of EUS-FNA for diagnosing pancreatic cancer have reported sensitivities of 80–90%, specificities of 90–100%, and accuracies of 85–90% (26, 27). To address these limitations, EUS-FNB was introduced in the early 2000s. It utilizes a different type of needle to procure tissue cores with higher diagnostic accuracy (24, 28, 29).

The cell block technique entails processing sediments, blood clots, or grossly visible tissue fragments from cytologic specimens into paraffin blocks, which can then be sectioned and stained using standard histopathologic methods (30). Besides serving as a supplement to other cytologic preparations for morphology evaluation by displaying tissue architecture, cell blocks are increasingly favored because they yield tissue sections suitable for further immunohistochemical and molecular studies. Employing cell block preparation alongside cytology has been shown to enhance the diagnostic accuracy of EUS-FNA, making it comparable to or better than FNB (31–34).

The primary aim of this study was to assess the effectiveness of EUS-FNA cytology in diagnosing the most common pancreatic lesions by comparing cytologic findings with tissue diagnoses obtained from Whipple procedures (the gold standard). In addition, we sought to explore how cell block utilization could further improve the diagnostic accuracy of EUS-FNA cytology in these cases.

## Material and Methods

### Study Design and Patients

This cross-sectional retrospective study was conducted in the pathology department of Firoozgar Hospital, Tehran, Iran, from January 2019 to December 2023. Firoozgar Hospital is a high-volume facility that acts as the gastrointestinal referral center for the Iran University of Medical Sciences. The study was designed following the Declaration of Helsinki after obtaining approval from the Ethics Committee of the Iran University of Medical Sciences (IR.IUMS.REC.1403.242). 

All the patients with EUS-FNA cytology specimens from the pancreatobiliary area and subsequent Whipple surgery throughout the five years were included in this study. The cases with insufficient clinical data, a history of pre-surgery chemoradiation , and deteriorated cytologic slides were excluded. Medical and pathologic documents were used to record the patients' characteristics, including age and gender, as well as the clinical data, such as tumor location (pancreatic head, body, and tail; common bile duct; and ampullary and periampullary), tumor size, puncture route of EUS-FNA, presence or absence of cell block, cytologic and pathologic diagnosis, and histologic tumor grade. Two experienced gastrointestinal pathologists reviewed the cytologic and pathologic slides, and the cytologic study's accuracy was determined by comparing it to a resection pathology diagnosis, which is the gold standard. 

### EUS Procedure

EUS operations were performed using an echo-endoscope (GF-UC140; Olympus America, Center Valley, Pennsylvania). The 22- or 25-gauge needle (Echotip; Boston Company, USA) was used for FNA and tissue collection. 

### Cytologic and Histopathologic Evaluation

All the cytologic slides were prepared and stained using the PAP and May Grunwald/Giemsa (MGG) stain procedures (35, 36). The cell blocks were prepared from the pellet of centrifuged fluid by adding plasma and thrombin to trap the cellular material in a clot and processing it as a formalin-fixed specimen. Hematoxylin and eosin were used to stain cell blocks and histology slides. Both histology slides and cell blocks underwent immunohistochemistry (IHC) in accordance with the manufacturer's instructions (Master Diagnostica, Spain). The Cytological and histological diagnosis was made using the Papanicolaou Society of Cytopathology (PSC) reporting system and the World Health Organization classification of 2019, respectively. Histomorphology was used for all the tumors to be graded, and for NETs, the ki67 labeling index was also applied. 

### Statistical Analysis

Diagnostic accuracy was defined as the percentage of lesions sampled that matched the final diagnosis. Continuous variables were presented as medians and ranges of values, whereas categorical variables were reported as proportions. Dichotomized variables were compared using Fisher’s exact 2-tailed test, and continuous variables were measured using a Mann-Whitney test. Statistical significance was determined a priori at P-value< 0 .05. 

## Results

**Fig. 1:**
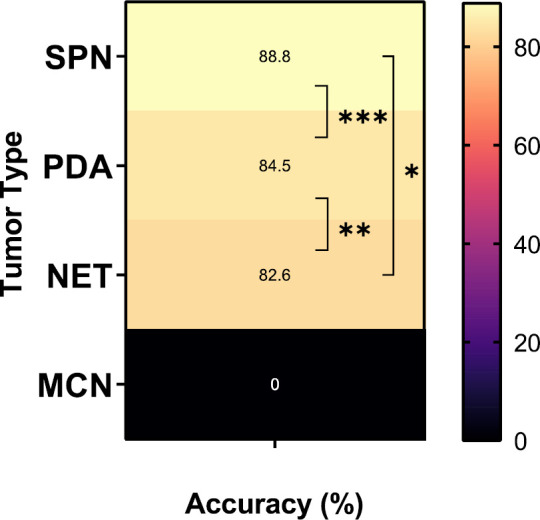
The cytology accuracy (%) of different epithelial tumors.

SPN: solid pseudopapillary neoplasm; PDA: pancreatic ductal adenocarcinoma; NET: neuroendocrine tumor; MCN: mucinous cystic neoplasm.

A total of 108 Whipple surgeries and distal pancreatomies were performed following EUS-FNA cytologic diagnoses of a neoplastic process. The total accuracy was 80%, sensitivity was 90%, and there was a less than 1% false-positive rate. IHC was performed in 22 cell blocks and 24 histology blocks to determine the tumor grade or to provide a definitive diagnosis. Seventy cases (65%) were PDAs, 23 cases (21.3%) were NETs, 9 cases (8.2%) were SPNs, 5 cases (4.6%) were MCNs, and one case (0.9%) was simply chronic pancreatitis. Overall, there was no significant difference in accuracy across the various epithelial tumors (Figure 1). 

The puncture routes were trans-duodenal in 83 instances (77%) and trans-gastric in 25 cases (23%). 56 (52%) patients were male, while 52 (48%) were female. Table 1 displays the clinicopathologic characteristics of cases according to their final diagnosis. 

A higher prevalence of PDA (*P*<0.0001) was noted in male patients, while a higher prevalence of NET (*P*=0.04) was observed in females. SPN and MCN were exclusively observed in females. 

**Fig. 2:**
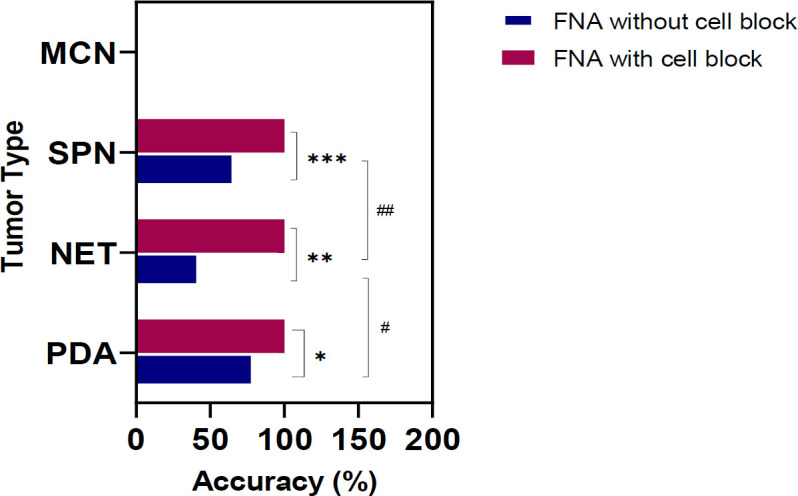
Comparison of the cytology accuracy between FNA and FNA with the cell block.

Cellblocks were present in 38 (35%) instances. Figure 2 compares the accuracy of cytology in the absence and presence of cell blocks in various epithelial tumors.

**Fig. 3:**
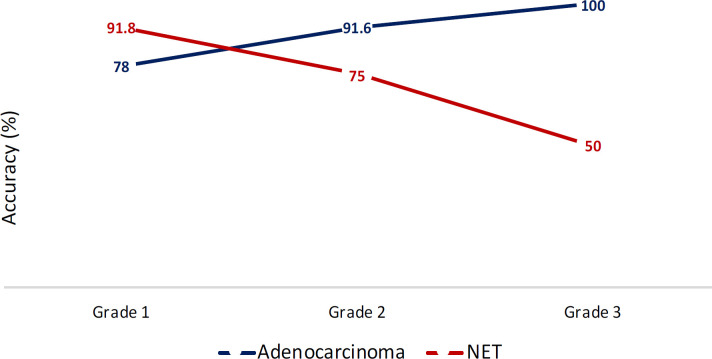
The accuracy of cytology without cell block for diagnosis of adenocarcinoma and NET based on the histologic grade.

Among the 16 cases correctly diagnosed as neuroendocrine tumors and had cell block, 5 (31%) showed misclassification for tumor grade in Ki-67 IHC and mitotic count. Figure 3 depicts the cytology accuracy in adenocarcinoma and NET based on histological grade.

**Fig. 4:**
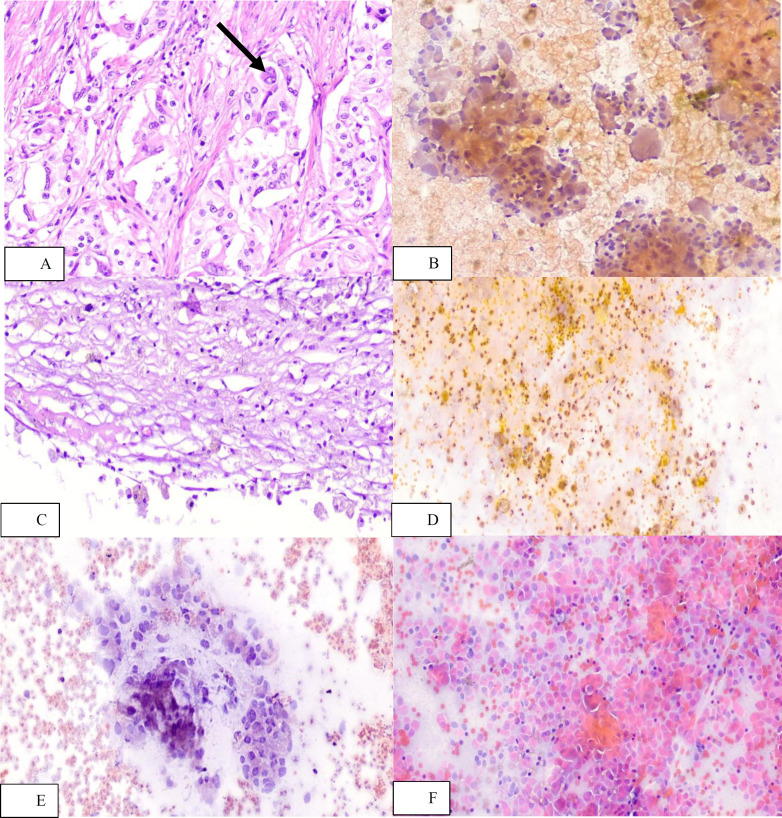
Ganglion cells (arrow) in a case of gangliocytic paraganglioma (A: H&E, x200) caused misdiagnosis of adenocarcinoma in cytology (B: PAP stain, x200). Focal degeneration and hemorrhage in a case of mucinous cystic neoplasm (C: H&E, x200) caused misdiagnosis of pseudocyst in cytology (D: PAP stain, x200). Degenerated cells in the cytology (E: PAP stain, x200) lead to misdiagnosis of adenocarcinoma in a case of neuroendocrine tumor, and conversely, minimal atypia and plasmacytoid epithelial cells (F: PAP stain, x200) caused misdiagnosis of neuroendocrine tumor in a case of adenocarcinoma.

In 21 cases, final pathologic diagnosis did not correspond to the FNA cytology result. Table 2 shoew the details of these cases as well as potential sources of discrepancies. Figure 4 depicts the cytology and pathology of some of the instances that had no cell block material.

∗*P*=0.66, ∗∗*P*= 0.83, ∗∗∗*P*= 0.73.

∗*P*<0.0001, ∗∗*P*<0.0001, ∗∗∗*P*=0.06, #*P*= 0.0008, ##*P*=0.22

MCN: mucinous cystic neoplasm; SPN: solid pseudopapillary neoplasm; NET: neuroendocrine tumor; PDA: pancreatic ductal adenocarcinoma.

NET: neuroendocrine tumor.

**Table 1:** Clinicopathologic characteristics of the cases.

Final Diagnosis	Number		Age range (mean) (y/o)		Sex				Tumor location*								
					Male (%)		Female (%)		Pancreas Head (%)		Pancreas Body (%)	Pancreas Tail (%)			CBD (%)	Ampullary- Periampullary (%)	
Adenocarcinoma	70		35-79 (59.3)		47 (67)		23 (33)		41 (59)		3(4)	2(3)			9 (13)	15 (21)	
NET	23		15-67 (47.8)		8 (35)		15 (65)		9(39)		5(22)	7(30)			0(0)	2 (9)	
SPN	9		11-40 (24.6)		0 (0)		9 (100)		6(67)		1 (11)	2(22)			0(0)	0 (0)	
MCN	5		24-61 (43.7)		0 (0)		5(100)		0(0)		3(60)	2(40)			0(0)	0 (0)	
Chronic pancreatitis	1		62		1 (100)		0 (0)		NA		NA	NA			NA	NA	
Total	108		11-79 (53.7)		56 (52)		52 (48)		56 (52)		11 (10)	14 (13)			9 (8)	18 (17)	
Final Diagnosis		Number		Age range (mean) (y/o)		Sex				Tumor size (mean) (cm)**			Gross				
						Male (%)		Female (%)					Solid (%)	Solid- Cystic (%)			Cystic (%)
Adenocarcinoma		70		35-79 (59.3)		47 (67)		23 (33)		0.3-13.5 (3.3)			68 (96)	1 (1)			2 (3)
NET		23		15-67 (47.8)		8 (35)		15 (65)		1.2-10 (4.3)			21 (91)	2 (9)			0 (0)
SPN		9		11-40 (24.6)		0 (0)		9 (100)		2.2-11 (4.8)			7 (78)	2 (22)			0 (0)
MCN		5		24-61 (43.7)		0 (0)		5(100)		1-16 (5.6)			0 (0)	0 (0)			5 (100)
Chronic pancreatitis		1		62		1 (100)		0 (0)		NA			NA	NA			NA
Total		108		11-79 (53.7)		56 (52)		52 (48)		0.3-16 (3.7)			97 (90)	5 (5)			6 (5)

NET: neuroendocrine tumor; SPN: solid pseudopapillary neoplasm; MCN: mucinous cystic neoplasm; NA: not applicable.

*Determined by EUS and gross examination of the Whipple specimen.

**The largest diameter of the mass was determined by the EUS and gross examination of the Whipple specimen.

**Table 2:** Details and likely sources of inaccuracy in the mismatched instances.

Case	Tumor site	Tumor size (mm)*	FNA cytology diagnosis	Final pathology diagnosis	Tumor Grade	Cell block	Possible Cause of the pitfall
1	Pancreas Head	20	Negative (Pseudocyst)	PDA	1	No	-absence of cell block -small sized mass -Adenocarcinoma arising on MCN
2	CBD	3.5	NET	PDA	1	No	-absence of cell block -very well differentiated adenocarcinoma with minimal cellular atypia
3	Pancreas Head	17	Negative	PDA	2	No	-absence of cell block -small sized mass -extensive necrosis in tumor
4	Pancreas Head	30	NET	PDA	1	No	-absence of cell block -very well differentiated adenocarcinoma with minimal cellular atypia
5	CBD	25	Negative	PDA	1	Yes	The cytology slides and the cell block were not representative of the mass.
6	Ampullary	10	Negative	PDA	2	No	-absence of cell block - small sized mass -Ampullary location of the mass
7	Pancreas Head	25	NET	PDA	1	No	-absence of cell block -very well differentiated adenocarcinoma with minimal cellular atypia
8	Periampullary	15	Negative	PDA	1	No	-absence of cell block - small sized mass -Periampullary location of the mass
9	Pancreas Head	40	NET	PDA	1	No	-absence of cell block - very well differentiated adenocarcinoma with minimal cellular atypia
10	Ampullary	25	NET	PDA	1	No	-absence of cell block -very well differentiated adenocarcinoma with minimal cellular atypia
11	Periampullary	11	PDA	GPG	1	No	-absence of cell block -The Ganglion cells were considered isolated cells with significant atypia in cytology.
12	Pancreas Head	60	PDA	NET	2	No	-absence of cell block -significant atypia and pleomorphism
13	Pancreas Head	35	Negative (Pseudocyst)	NET	2	No	-absence of cell block -cystic degeneration and hemorrhage in the mass
14	Pancreas Tail	95	SPN	NET	2	No	-absence of cell block -Papillary architecture
15	Pancreas Body	42	NET	SPN	NA	No	-absence of cell block -scant papilla in cytology
16	Pancreas Body	60	Negative	MCN	Low grade	No	-No mucinous material or mucin cell were seen in cytology.
17	Pancreas Body	48	Negative	MCN	Low grade	No	-No mucinous material or mucin cell were seen in cytology.
18	Pancreas Body	28	Negative	MCN	Low grade	No	-No mucinous material or mucin cell were seen in cytology.
19	Pancreas Body	35	Negative	MCN	Low grade	No	-No mucinous material or mucin cell were seen in cytology.
20	Pancreas Body	40	Pseudocyst	MCN	Low grade	No	-No mucinous material or mucin cell were seen in cytology.-Hemorrhage
21	NA	NA	PDA	Chronic pancreatitis	NA	No	-Absence of cell block -Reactive atypia

*: Greatest diameter

PDA: pancreatic ductal adenocarcinoma; CBD: common bile duct; NET: neuroendocrine tumor; SPN: solid pseudopapillary neoplasm; MCN: mucinous cystic neoplasm; NA: not applicable.

## Discussion

In this study, EUS-FNA cytology achieved an overall accuracy of 80% and a sensitivity of 90% across 108 cases, with accuracy rising to 100% when cell blocks were available. These findings align with previous studies, such as those by Kusunose et al., which reported 94.1% sensitivity and 96.8% accuracy in 31 cases using EUS-FNA and cell blocks (37–39). However, when tumor type and histologic differentiation were considered, the results varied. In a subgroup analysis by tumor type, FNA cytology was more effective for detecting PDA and SPN than NET, especially in the absence of cell blocks. Furthermore, in PDA, lower tumor grade correlated with higher cytology accuracy; conversely, in NET, poor differentiation diminished cytology accuracy.

PDAs can be recognized cytologically by their smear pattern and cytomorphological features, typically presenting scattered glandular clusters and individual cells. By contrast, NETs often show a uniform, solid cellular smear appearance. Two extremes of tumor differentiation may be misdiagnosed: well-differentiated adenocarcinoma might be mistaken for NET, whereas a poorly differentiated NET or neuroendocrine carcinoma could be confused with adenocarcinoma. The absence of a cell block slide precludes ancillary testing that might otherwise clarify the diagnosis. Recent findings also indicate that using cell blocks or FNB improves diagnostic accuracy (28, 40). SPN is a well-characterized epithelial pancreatic tumor of uncertain histogenesis and exhibits histologic similarities to NET, necessitating IHC testing for accurate diagnosis. Consequently, EUS-FNA cytology samples without cell blocks or with limited cellularity can be misdiagnosed as NET, and vice versa (1). None of the MCNs in this study were correctly identified by cytology because of insufficient mucinous material and mucin cells; however, applying the current WHO reporting system—which includes elevated cyst fluid CEA levels as indicative of MCN—would have led to correct diagnoses in most cases (41, 42).

Regarding tumor grade in NETs, undegrading with Ki-67 IHC may occur in cell blocks, as observed in our study. Nevertheless, other investigations have shown equivalent results between cell block samples and surgical specimens, suggesting that differences in sample characteristics and IHC techniques are two possible pitfalls (43–45).

In our series, all tumor types except MCN were more commonly located in the pancreatic head. Additionally, our incidence rates for PDA, MCN, and SPN in males and females were consistent with earlier studies; however, contrary to previous findings, NET was more frequent in females (42, 46–49).

The primary causes of sampling errors leading to cytologic pitfalls in diagnosing adenocarcinomas were tumor locations outside the pancreatic head (including CBD, ampullary, and periampullary regions), smaller tumor size (≤20 mm), tumor necrosis, and very well-differentiated lesions. Common diagnostic challenges for NETs included cystic degeneration, papillary architecture, pleomorphism, and ganglion cells. Similar factors, such as necrosis, hemorrhage, inflammation, and contaminating gastrointestinal epithelial cells, have been documented in previous research as notable pitfalls in the cytologic diagnosis of pancreatic neoplasms (29, 50).

Pathologists should be aware of these potential pitfalls to improve EUS-FNA cytology accuracy for pancreatobiliary lesions and optimize patient management. Additionally, preparing cell blocks from cytology material would be beneficial.

The strengths of our study include the use of radical surgery pathology as the gold standard and our analysis of the causes of diagnostic pitfalls. However, we lacked follow-up data for patients with negative or benign cytologic results, preventing us from assessing specificity. Moreover, although this study compared multiple epithelial pancreatobiliary tumors, the sample size was small, especially for NET, SPN, and MCN. Consequently, further research is recommended to clarify the role of cytology in diagnosing these epithelial tumors.

## Conclusion

Our findings indicate that the likelihood of an accurate diagnosis increases when cytology is combined with a cell block. Additionally, a higher tumor grade in adenocarcinoma and a lower tumor grade in organoid-structured tumors, such as NET and SPN, further enhance diagnostic accuracy. 
